# Brain Abnormalities in Congenital Fibrosis of the Extraocular Muscles Type 1: A Multimodal MRI Imaging Study

**DOI:** 10.1371/journal.pone.0133473

**Published:** 2015-07-17

**Authors:** Wen Miao, Fengyuan Man, Shaoqin Wu, Bin Lv, Zhenchang Wang, Junfang Xian, Bernhard A. Sabel, Huiguang He, Yonghong Jiao

**Affiliations:** 1 State Key Laboratory of Management and Control for Complex Systems, Institute of Automation, Chinese Academy of Sciences, Beijing, China; 2 Department of Radiology, Beijing Tongren Hospital, Capital Medical University, Beijing, China; 3 Beijing Tongren Eye Centre, Beijing Tongren Hospital, Capital Medical University, Beijing Ophthalmology and Visual Science Key Lab, Beijing, China; 4 Department of Radiology, Beijing Anzhen Hospital, Capital Medical University, Beijing, China; 5 China Academy of Telecommunication Research of Ministry of Industry and Information Technology, Beijing, China; 6 Beijing Friendship Hospital, Capital Medical University, Beijing, China; 7 Otto-von-Guericke University of Magdeburg, Medical Faculty, Institute of Medical Psychology, Magdeburg, Germany; Institute of Psychology, Chinese Academy of Sciences, CHINA

## Abstract

**Purpose:**

To explore the possible brain structural and functional alterations in congenital fibrosis of extraocular muscles type 1 (CFEOM1) patients using multimodal MRI imaging.

**Methods:**

T1-weighted, diffusion tensor images and functional MRI data were obtained from 9 KIF21A positive patients and 19 age- and gender- matched healthy controls. Voxel based morphometry and tract based spatial statistics were applied to the T1-weighted and diffusion tensor images, respectively. Amplitude of low frequency fluctuations and regional homogeneity were used to process the functional MRI data. We then compared these multimodal characteristics between CFEOM1 patients and healthy controls.

**Results:**

Compared with healthy controls, CFEOM1 patients demonstrated increased grey matter volume in bilateral frontal orbital cortex and in the right temporal pole. No diffusion indices changes were detected, indicating unaffected white matter microstructure. In addition, from resting state functional MRI data, trend of amplitude of low-frequency fluctuations increases were noted in the right inferior parietal lobe and in the right frontal cortex, and a trend of ReHo increase (p<0.001 uncorrected) in the left precentral gyrus, left orbital frontal cortex, temporal pole and cingulate gyrus.

**Conclusions:**

CFEOM1 patients had structural and functional changes in grey matter, but the white matter was unaffected. These alterations in the brain may be due to the abnormality of extraocular muscles and their innervating nerves. Future studies should consider the possible correlations between brain morphological/functional findings and clinical data, especially pertaining to eye movements, to obtain more precise answers about the role of brain area changes and their functional consequence in CFEOM1.

## Introduction

**Fig 1:**
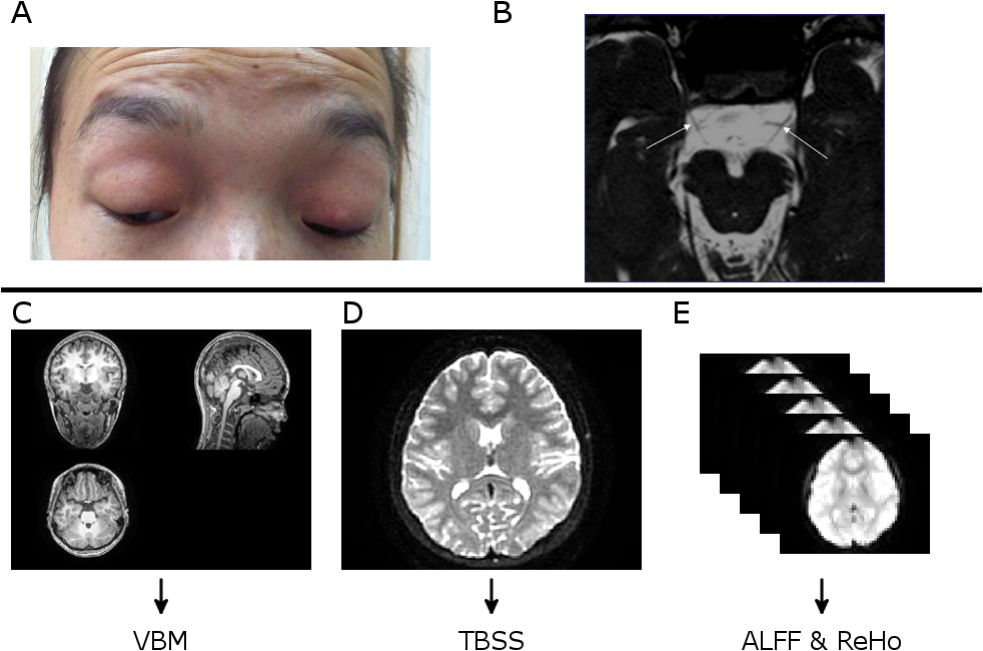
The research strategy and experiment design. A, Phenotypes of a typical patient with CFEOM1; B, CNIII (Oculomotor nerve) hypoplasia shown in MRI (arrows) of this typical patient with CFEOM1 which were used to identify the atrophy of extraocular muscle. The structural abnormality of optic nerve and extraocular muscles have been well studied in CFEOM. We then focus on the cerebral alterations associated with CFEOM; C, T1-weighted MRI scanned from the patient, shown with three different views. T1 data were later feed into VBM processing pipeline; D, The DTI images scanned from the patient. DTI date were then used in TBSS processing; E, The resting—state fMRI data from this CFEOM1 patient. ALFF and ReHo processing were then carried out using the resting-state fMRI data.

Congenital Fibrosis of the Extraocular Muscles (CFEOM) refers a group of congenital /hereditary strabismus syndromes characterized by congenital non-progressive ophthalmoplegia with or without ptosis[1]. Patients with CFEOM may show rapid convergent eyes movement on attempted up gaze, simulating convergence retraction nystagmus [2]. Based on clinical performance, mainly three types of CFEOM have been identified, of which CFEOM type1 (CFEOM1) is the ‘classic’ and most common type [3]. Generally, patients with CFEOM1 are accompanied by congenital bilateral blepharoptosis and ophthalmoplegia, with the eyes partially or completely fixed in infraduction [4] (Fig 1A). Although it is rather uncommon with a prevalence of 1/230,000 [5], this eye movement disease can cause severe problems such as the poor appearance, impairment of visual acuity and binocular vision, and easily noticeable psychosocial problems [2, 4].

CFEOM was previously considered to be caused by primary muscle pathology, but postmortem studies of a single CFEOM patient revealed abnormality of the alpha motor neurons of the oculomotor nucleus. Here, decreased numbers of motor neurons were found in oculomotor subnuclei which innervate the extraocular muscles and receive input from cerebral cortex [4]. In addition, genes necessary for the normal development and connectivity of brainstem ocular motoneurons are known to be mutated [6], which may explain the results obtained by magnetic resonance imaging (MRI) studies [7]. Based on these evidences, Assaf suggested that CFEOM is actually a neurological disorder [8], arising from an abnormal development of individual or multiple cranial nerve nuclei or their axonal connections [9]. Indeed, Then Cheng et al also concluded that CFEOM1 is a primary error in cranial nerve development [10].

Eye movements are initiated by cerebral cortical activity which acts on ocular motor control structures beyond the ocular reflexes. [11] While the mechanism of gene mutations and its relation to dysfunction of extraocular muscles and nuclei have been well studied in CFEOM, [6, 12–14] there are very few reports regarding possible changes in cerebral cortical areas related to eye movement control and the voluntary saccades circuit [15]. It is therefore still unclear whether and how brain structural and functional alterations occur in CFOEM patients.

Recent technical improvements in MRI make it possible to investigate the brain structural and functional development and their disease-associated alterations in a quantitative manner. Structural MRI studies using voxel-based morphometry (VBM) have been conducted to detect the local concentration changes of gray matter (GM) [16] between groups [17]. Compared with the conventional region-of-interest (ROI) analysis, the VBM methods is fully automated and unbiased voxel-wise approach which is not restricted to specific brain regions. Diffusion tensor imaging (DTI) is another MR imaging technique which is capable of providing some measures that are sensitive to white matter (WM) structure changes, e.g. fractional anisotropy (FA), mean diffusivity (MD), etc. Based on these measurements, tract-based spatial statistics (TBSS) was recently developed to evaluate the whole brain WM alterations in various diseases [18]. In addition, with resting state functional MRI, investigators can characterize the brain spontaneous functional activities with some local features, e.g. the amplitude of low-frequency fluctuations (ALFF)[19] and regional homogeneity (ReHo)[20], etc. Besides their wide application in neurodegenerative diseases [21–23], these multimodal imaging techniques have also been used to explore the brain structure and function in eye-related diseases, e.g. strabismus and amblyopia [24, 25] etc. However, due to the low incidence of CFEOM, no multimodal MRI study has been conducted for a larger group of CFEOM patients; hence little is known about their possible brain structural and functional alterations.

In the present study, we aimed to explore the possible brain alterations in a group of CFEOM1 patients using structural MRI, DTI and resting state functional MRI. Some quantitative assessments, including VBM, TBSS and ALFF [19] and ReHo [20], were conducted on these multimodal MRI imaging. We hypothesized that there might be structural and functional alterations in cerebral cortical areas that are related to the abnormalities of the extraocular muscles in patients with CFEOM1.

## Materials and Methods

### Participants

Nine KIF21A-mutation positive patients with classic CFEOM1 (age at imaging [mean ± std]: 24.3±9.9 yrs; range: 15 ~ 49 yrs; 1 male) and 19 age and gender matched (age at imaging [mean ± std]: 25.3 ± 9.6 yrs; range: 16 ~ 53 yrs, 3 males) healthy controls were included in this study. All subjects were all right handed. They underwent the ophthalmic examination including corrected visual acuity, ocular motility, measurement of palpebral fissure height and levator function, binocular alignment, anterior segment anatomy, and ophthalmoscopy. All 9 patients with CFEOM1 had blepharoptosis. Ocular alignment was evaluated in all positions of gaze, and ophthalmic histories were also obtained. By checking with orbital and intracalvarium MRI, in all the patients hypoplasia of the ocular motor nerves and the extraocular muscles could be shown (Table 1). The abducens nerves could not be visualized in MRI in 7 out of 9 patients, in 3 cases it could not be visualized bilaterally and 4 cases unilaterally. The healthy controls were enrolled by excluding any ophthalmic or neurological diseases which could affect the visual pathway and brain structure.

**Table 1:** Clinical details of the patients.

Patient No/Gender/Age (yr)	Corrected Visual Acuity(LogMAR): Right/Left	Horizontal Alignment	MRI (Orbit)	MRI (Cistern Segment)
1/F/24	0.2/0.3	XT	hypoplasia of SR, LPS, IR bilaterally; left MR	Hypoplasia of CNIII bilaterally
2/M/26	0.4/0.3	XT	hypoplasia of SR, LPS, MR, IR bilaterally	Hypoplasia of CNIII bilaterally, absence of left CNVI
3/F/18	0.2/0.09	XT	hypoplasia of SR, LPS, MR, IR bilaterally; left LR	Hypoplasia of CNIII bilaterally
4/F/22	0.3/0.2	XT	hypoplasia of SR, LPS, MR, IR, LR bilaterally	Hypoplasia of CNIII bilaterally, absence of left CNVI
5/F/23	0.4/0.5	XT	hypoplasia of SR, LPS, MR, IR, LR bilaterally	Hypoplasia of CNIII bilaterally, absence of CNVI bilaterally
6/F/15	0.25/0.2	XT	hypoplasia of SR, LPS, MR, IR, LR bilaterally	Hypoplasia of CNIII bilaterally
7/F/18	0.2/0.2	Orthotropic	hypoplasia of SR, LPS, MR, IR bilaterally	Hypoplasia of CNIII bilaterally, absence of right CNVI
8/F/49	0.3/0.48	XT	hypoplasia of SR, LPS, MR, IR bilaterally	Hypoplasia of CNIII bilaterally, absence of right CNVI
9/F/24	0.4/0.2	XT	hypoplasia of SR, LPS, MR, IR bilaterally	Hypoplasia of CNIII bilaterally, absence of CNVI bilaterally

XT, exotropia; SR, superior rectus; IR, inferior rectus; MR, medial rectus; LR, lateral rectus; LPS, levator palpabrae superioris; CNIII, Oculomotor nerve; CNVI, Abducens nerve. All patients had bilateral blepharoptosis, limited supraduction, and essentially complete ophthalmoplegia.

The study was approved by Beijing Tongren Hospital’s review board, and written informed consent was obtained from all the subjects or their guardians according the Declaration of Helsinki. The individual in this manuscript has given written informed consent (as outlined in PLOS consent form) to publish these case details.

### MRI Data Acquisition

All MRI scans were performed on a 3T HDxt MR imaging scanner (Signa HDxt, GE Healthcare, Milwaukee, Wisconsin) using a 8-channel phased-array head coil. Three types of images were acquired for each subject, including T1-weighted structural, DTI and resting state fMRI images. T1-weighted structural images were obtained using GE’s BRAVO sequence (IR-prep, fast SPGR with parameters tuned to optimize brain tissue contrast) with the following parameters: repletion time (TR) = 9 ms; echo time (TE) = 3.5 ms; inversion time = 450 ms; flip angle = 13°; field of view (FOV) = 24×24 cm; acquisition matrix = 256×256; and slice thickness = 1 mm (Fig 1C). DTI Images were acquired using spin-echo, echo-planar imaging sequence with the following imaging parameters: TR/TE = 17,000/93 ms, acquisition matrix = 256×256, FOV = 24×24 cm, slice thickness = 2mm, and no intersection gap. Motion-probing gradients were applied along 15 non-collinear directions with a b factor of 1000 s/mm^2^ after an acquisition without diffusion weighting (b = 0 s/mm^2^) for reference (Fig 1D). Resting-state fMRI images were acquired using echo-planar imaging sequence with the following parameters: TR/TE = 2000/35 ms, flip angle = 90°, acquisition matrix = 64 × 64, FOV = 24 × 24 cm, slice thickness = 4 mm, gap = 1 mm, voxel size = 3.75 × 3.75 × 5 mm. Resting-state scans lasted for 400 s to collect 200 volumes for each subject. Subjects were instructed to lay still and awake with their eyes closed during the resting state fMRI scan (Fig 1E).

### Image Processing and Statistical Analysis

All images were reoriented to match the orientation of the MNI152 standard template images and processed off-line as follows:

T1-weighted structural images were analyzed using FSL-VBM framework (http://fsl.fmrib.ox.ac.uk/fsl/fslwiki/FSLVBM) [17]. First, non-brain tissue pixels were removed [26] and brain tissues were segmented into GM, WM and CSF in native space [27]. The segmented GM images were then non-linearly registered to the GM ICBM-152 template [28] and then averaged to create a study-specific GM template. All the native GM images were then non-linearly registered to the GM template and modulated to correct for local expansion or contraction. The modulated images were subsequently smoothed with an isotropic Gaussian kernel with a standard deviation of 3 mm. At last, permutation-based non-parametric testing (10,000 permutations) was used in a voxel-wise general linear model for comparison of patients versus normal controls [29]. Threshold-free cluster enhancement (TFCE)[30] method was used for multiple comparisons to identify cluster-like structures The statistical threshold was p < 0.05.

For each subject, fifteen DTI volumes with b value of 1000 s/mm^2^ were affine registered to the b0 volume for correction of eddy current distortion and simple head motion. Non-brain voxels were removed and a fractional intensity threshold of 0.3 was selected to generate a brain-extracted 4D image and a binary brain mask, which were used for fitting diffusion tensor model at each voxel [31]. Then some DTI’s measures were calculated including fractional anisotropy (FA), mean diffusivity (MD) axial diffusivity (AD) and radial diffusivity (RD). The standard TBSS procedure [18, 32, 33] was then applied on these DTI’s measures to generate the skeleton images. Voxel-wise statistical analysis of individual skeleton images of CFEOM1 patients versus normal controls was performed using a nonparametric permutation test [29]. And TFCE[30] method was used for multiple comparisons in order to identify significantly changed clusters. The statistical threshold was p < 0.05.

For resting-state fMRI images, we selected ALFF [19] and ReHo [20] methods to characterize the amplitude and the synchronization of the local spontaneous brain activity, respectively. The fMRI image processing was carried out using SPM8 (www.fil.ion.ucl.ac.uk/spm/) and the REST software [34]. The first ten volumes of individual resting state fMRI data were discarded. The remaining volumes were realigned to the first one to correct for head motion. The individual fMRI images were then spatially normalized to the standard template and re-sampled to 3×3×3 mm voxel size. The linear trends were regressed and a band-pass filter were applied at 0.01~0.08 Hz. ALFF value on each voxel was calculated by averaging the square root of power spectrum from 0.01 Hz to 0.08 Hz [19], and then standardized by dividing the global mean ALFF value. ReHo value on each voxel was obtained by calculating the Kendall’s coefficient of concordance (KCC) within a cubic cluster size of 27 voxels [20]. Finally, two sample t-tests were applied to investigate the possible differences of these two indexes between patients with CFEOM1 and healthy controls.

## Results

**Fig 2:**
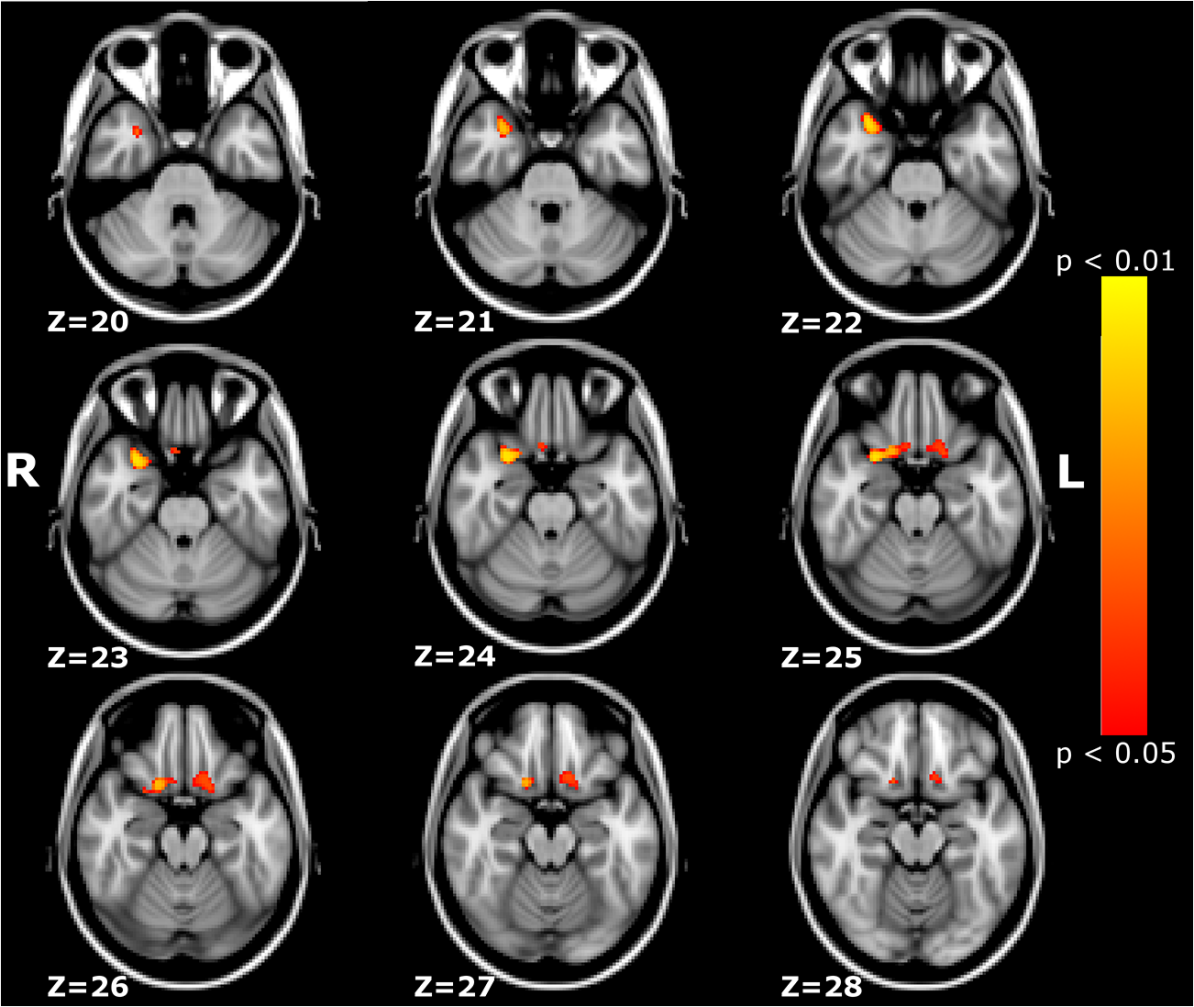
VBM results. Brain areas showing significant GM increases, including the right temporal pole and bilateral orbital frontal cortex (p < 0.05, corrected for multiple comparisons).

VBM analysis on structural MRI revealed that CFEOM1 patients had significant GM increases in brain regions including the right temporal pole and bilateral orbital frontal cortex (Table 2) (p < 0.05, TFCE corrected). Fig 2 illustrates their positions on the MNI standard template.

**Table 2:** Brain areas with regional gray matter changes.

Anatomical location	Cluster size (voxels)	MNI-Space (mm; X, Y, Z)	p-Value
R-FOC, R-TP	229	(30, -12, -26)	0.005
L-FOC	89	(-16, 14, -20)	0.035

Voxel size = 2×2×**2** mm; R = right; L = left; FCO = Frontal Orbital Cortex; TP = Temporal Pole.

No significant alterations were detected in any DTI measures (FA, MD, AD and RD) between the CFEOM1 group and healthy group.

**Fig 3:**
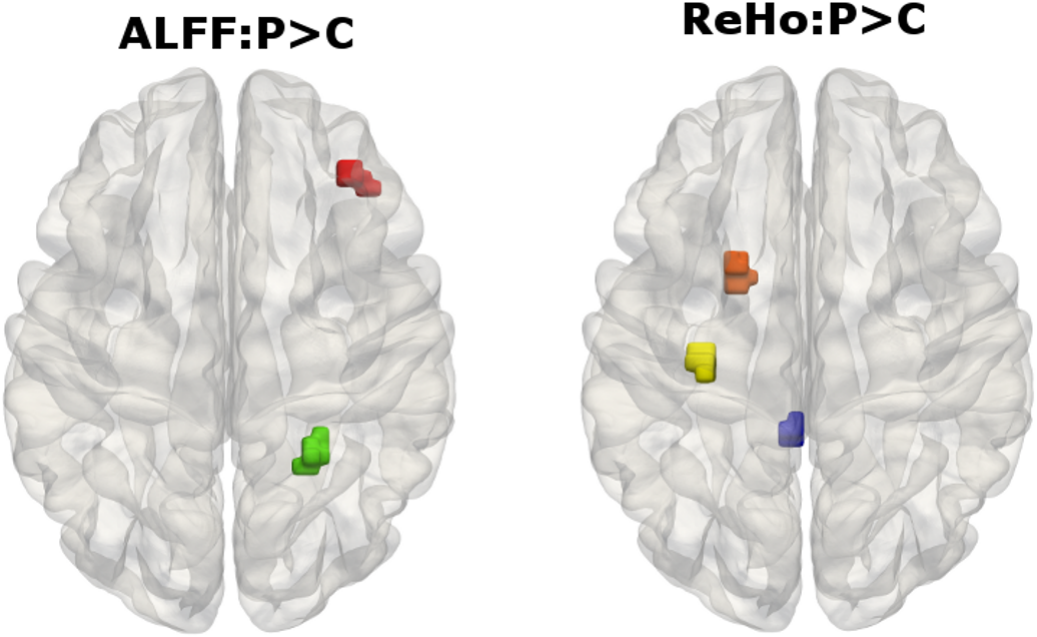
fMRI data results. ALFF increase (p< 0.001, uncorrected) in right inferior parietal lobe and right frontal cortex and ReHo increase (p<0.001 uncorrected) in left precentral gyrus, left orbital frontal cortex, temporal pole and Cingulate gyrus.

With resting state MRI, we did not detect signifcant changes in ALFF, or ReHo after multiple correction. However, when compared with healthy controls, we found that the patients with CFEOM1 showed a trend of ALFF increase (p< 0.001, uncorrected) in the right inferior parietal lobe and right frontal cortex and also a trend of ReHo increase (p<0.001 uncorrected) in the left precentral gyrus, left orbital frontal cortex, temporal pole and the posterior division of cingulate gyrus (Table 3 and Fig 3).

**Table 3:** Functional MRI results.

	Cluster size (voxels)	MNI-Space (mm; X, Y, Z)	p-Value
**ALFF: CFEOM > Controls**	950	(24, -48, 33)	0.000
	737	(39, 36, 0)	0.000
**ReHo: CFEOM > Controls**	950	(-27, -21, 60)	0.000
	800	(-24, 9, -14)	0.000
	725	(-3, -42, 3)	0.000

## Discussion

In the present study, a multimodal MRI imaging strategy was employed to investigate possible brain abnormalities in patients with CFEOM1. Quantitative analysis methods were applied to characterize the image features of structural, diffusion tensor and functional MRI. Our study demonstrated, for the first time, that there were some brain structural and functional alterations associated with CFEOM1. These alterations were documented with the significant GM changes detected by VBM analysis of structural MRI, and the slight change in spontaneous brain activity revealed by fMRI indices. However, TBSS analysis on DTI revealed the white matter microstructure of in patients with CFEOM1 was unaffected.

With genetic techniques, patients with CFEOM1 were identified to be accompanied by the heterozygous missense mutations in KIF21A [6, 10]. Although the potential role of KIF21A in brain development is still unknown, KIF21A expression were found to be widely distributed in many neuronal populations of the central and peripheral nervous system from early development into maturity [35]. CFEOM1 has been established to be a primary error in cranial nerve development [10]. To obtain a good physiological rationale to better understand the basis of CFEOM1, we now scouted more attention to look for the possible secondary changes in the brain regions of patients with CFEOM1.

The temporal pole is believed to play a role in integrating visual information and viscero-autonomic responses, and may modulate the vestibular system to reduce or enhance the level of vestibular control over eye movements [36–38]. The CFEOM1 patients showed increased GMV in the right temporal lobe, reflecting increased multisensory integration to support visual task, such as eye movement control and visual identification. The orbital frontal cortex is one of the least understood areas of cerebral cortex [39]. Previous studies suggest this area to be a component of brain systems critically engaged in memory, reward and decision-making mechanisms. Furthermore, it is particularly affected in various mental and neurological disease, such as major depression [40], Tourette syndrome [41] and dementia [42]. However, unlike these neurodegenerative disorder patients who have GMV decreases in the orbital frontal cortex, CFEOM1 patients showed GMV increases in the orbital frontal cortex, indicating the functions of orbital frontal cortex were not impaired but strengthened. ReHo increases were also observed in the orbital frontal cortex, and temporal pole. This is consistent with the GMV changes in these areas.

DTI is becoming increasingly popular for its high sensitivity in detecting WM micro-structural alterations [43–45]. When analyzed with TBSS [18], DTI studies have the advantages of higher spatial registration and smoothing, thus enabling more accurate results. Interestingly, no significant difference in FA, MD AD or RD was detected in CFEOM1 patients by using TBSS. The absence of any differences suggests that the fiber myelination in identified WM areas (FA threshold of 0.2 was selected as the boundary of WM and GM) and the white matter connectivity pattern in the CFEOM1 patient group was not affected by the disease. However, the observed abnormality in oculomotor nucleus in previous studies suggests a decreased input/output in neural stimuli between these nuclei and cerebral cortex, indicating decreased brain integrity and altered eye movement control output patterns to the target muscles [4, 46]. The unchanged WM diffusion indices may suggest that the decreased input/output’s efferts on the brain structural and function is more subtle than expected. Thus, further studies are needed to resolve the question how the integrity of the eye movement related brain areas are altered in CFEOM1 patients.

Our study has some limitations and the findings should be interpreted with some caution. Firstly, we did not collect functional measures of eye movement. Secondly, we had a small sample size (n = 9) because of the low incidence of the disease, which is perhaps underpowered to detect more subtle changes. Finally, the potential role of KIF21A in brain development is unknown. Whether it will affect some other subgroup of nerves in other parts of brain is yet to be determined. Thus, our study should be viewed only as an exploratory step towards characterizing brain pathology and understanding the brain-based mechanisms of CFEOM1.

In conclusion, by studying a group of CFEOM1 patients using automated MRI voxel based morphometry, tract based spatial statists, and fMRI indices statists methods, we found that the patients showed alterations in cerebral cortex areas, which were not documented in previous cases or family based studies. These alterations indicated that the patient’s brain functions may have changed accordingly. Future studies should consider possible correlations between brain morphological/functional findings and clinical data, especially pertaining to eye movements, and to obtain more precise answers about the role of different brain area changes and their functional consequence in CFEOM1.
